# Supplementary material for: Dietary Cinnamon Bark Affects Growth Performance, Carcass Characteristics, and Breast Meat Quality in Broiler Infected with Eimeria tenella Oocysts
Source: Animals (Basel). 2022 Jan 11;12(2):166. doi: 10.3390/ani12020166 (PMC8772541; doi:10.3390/ani12020166)
Supplement: Supplementary file 1 [file animals-12-00166-s001.zip › animals-1445583-supplementary.pdf]

SUPPLEMENTARY TABLES

Supplementary Table S1. Ingredients and calculated nutrients of broilers starter and finisher diets.

Ingredient	Period	
	Starter	Finisher
Yellow corn	53.218	58.09
Soybean meal	37.85	32.15
Wheat bran	2.00	2.2
Corn gluten meal	1.4	0
Choline chloride CL 60	0.05	0.05
Corn oil	1.5	4.2
Dicalcuim phosphate DCP	1.98	1.615
Ground limestone	0.9	0.79
Salt	0.400	0.30
DL-methionine	0.292	0.25
Lysine-HCL	0.21	0.105
Vitamin–mineral premix[1]	0.200	0.200
Total	100	100
Metabolic energy, kcal/kg	3000	3200
Crude protein, %	23.0	20.0
Non phytate P, %	0.48	0.405
Calcium, %	0.96	0.81
D-lysine, %	1.28	1.06
Sulfur amino acids, %	0.95	0.83
Threonine, %	0.86	0.71

[1] The vitamin-mineral premix components per kg: vitamin A, 12000000 IU; vitamin D3, 5000000 IU; vitamin E, 80000 IU; vitamin K3, 3200 mg; vitamin B1, 3200 mg; vitamin B_2, 8600 mg; vitamin B_3, 65000 mg; pantothenic acid, 20000 mg; vitamin B_6, 4300 mg; biotin 220 mg; antioxidant(BHA+BHT), 50000 mg; B_9, 2200 mg; B_{12}, 17 mg; copper, 16000 mg; iodine, 1250 mg; iron, 20000 mg; manganese, 120000 mg; selenium, 300 mg, and zinc, 110000 mg.
